# Detection of acute pulmonary embolism using native repeated magnetic resonance imaging acquisitions under free-breathing and without respiratory or cardiac gating. A diagnostic accuracy study

**DOI:** 10.1016/j.ejro.2024.100558

**Published:** 2024-03-05

**Authors:** Koshiar Medson, Roberto Vargas Paris, Alexander Fyrdahl, Peder Wiklund, Sven Nyren, Eli Westerlund, Peter Lindholm

**Affiliations:** a Department of Physiology and Pharmacology, Karolinska Institutet, Stockholm SE-171 77, Sweden; b Department of Radiology, Division of chest imaging, Brigham and women’s Hospital, Boston, USA; c Department of Imaging and Physiology, Karolinska University Hospital, Stockholm SE-171 76, Sweden; d Department of Molecular Medicine and Surgery, Karolinska Institutet, Stockholm SE-182 88, Sweden; e Department of Radiology, Region Halland, Sweden; f Department of Clinical Sciences, Karolinska Institutet, Stockholm SE-182 88, Sweden; g Danderyd Hospital, Stockholm, Sweden; h Department of Emergency Medicine, University of California San Diego, USA; i Department of Clinical Physiology, Karolinska University Hospital

**Keywords:** Venous thromboembolism, Pulmonary embolism, Computed tomography angiography, Magnetic resonance imaging

## Abstract

**Objectives:**

Computed tomography pulmonary angiography (CTPA) is the gold standard diagnostic method for patients with suspected pulmonary embolism (PE), but it has its drawbacks, including exposure to ionizing radiation and iodinated contrast agent. The present study aims to evaluate the diagnostic performance of our in-house developed non-contrast MRI protocol for PE diagnosis in reference to CTPA.

**Methods:**

107 patients were included, all of whom underwent MRI immediately before or within 36 hours after CTPA. Additional cases examined only with MRI and a negative result were added to reach a PE prevalence of approximately 20%. The protocol was a non-contrast 2D steady-state free precession (SSFP) sequence under free-breathing, without respiratory or cardiac gating, and repeated five times to capture the vessels at different breathing/cardiac phases. The MRIs were blinded and read by two radiologists and the results were compared to CTPA.

**Results:**

Of the 243 patients included, 47 were positive for PE. Readers 1 and 2 demonstrated 89% and 87% sensitivity, 100% specificity, 98% accuracy and Cohen’s kappa of 0.88 on patient level. In the per embolus comparison, readers 1 and 2 detected, 60 and 59/61 (98, 97%) proximal, 101 and 94/113 (89, 83%) segmental, and 5 and 2/32 (16, 6%) subsegmental emboli, resulting in 81 and 75% sensitivity respectively.

**Conclusion:**

The repeated 2D SSFP can reliably be used for the diagnosis of acute PE at the proximal and segmental artery levels.

## Highlights

• The protocol was a 2D non-contrast steady-state free precession (SSFP) sequence under free-breathing, without respiratory or cardiac gating, and repeated five times to capture the vessels at different breathing and cardiac phases.
• The examination time was less than 10 minutes.
• The readers demonstrated a sensitivity of 87 and 89%, specificity of 100%, accuracy of 97 and 98% with a kappa value of 0.88 at patient level.

## 1 Introduction

Pulmonary embolism (PE) is the third most common cause of death among cardiovascular diseases, with an incidence of approximately 39–115 cases per 100,000 in Europe.[1], [2]

Due to its nonspecific signs and symptoms, the mainstay of diagnosis is imaging. Computed tomography pulmonary angiography (CTPA) is the recommended first-line modality as it has high sensitivity (83%) and specificity (96%), is readily available and has a short acquisition time of only a few seconds.[1]

However, CTPA has its drawbacks, including: exposure to ionizing radiation and the increased malignancy risk [3]the need for an iodinated contrast agent; and the associated risk for contrast-induced nephropathy and severe allergic reactions [4] and overuse.[5]

Attempts have been made to use magnetic resonance imaging (MRI) both with and without gadolinium contrast agent for PE diagnosis with varying results. The main issues have been the relatively long acquisition time, low spatial resolution, and in particular, the technical difficulties (Mainly timing) associated with the use of gadolinium contrast agent as demonstrated in PIOPED III.[6] These factors, as well as the relatively low availability, may explain why MRI is not among the recommended diagnostic procedures in the guidelines for diagnosing PE.[1]

The method outlined in the present study is an non-contrast 2D SSFP sequence under free-breathing and without respiratory or cardiac gating, repeated five times. It has been used in the clinical setting at our tertiary teaching hospital since 2012 for patients with suspected acute PE and contraindications to CTPA. The preliminary study describing this method was published in 2017 and showed a specificity of 100% for two independent/experienced readers and a sensitivity of 90% and 93%, respectively. Inter-reader agreement using Cohen's kappa was 0.97.[7]

The aim of the present study was to evaluate the accuracy of our in-house developed MRI protocol for detecting pulmonary embolism.

## 2 Materials & methods

### 2.1 Design and setting

A diagnostic accuracy study that included written informed consent from all subjects and was approved by the local ethics committee.

### 2.2 Patients

Patients were recruited between February 2012 and June 2020 from three tertiary hospitals. All CTPAs were performed according to the local clinical protocols at these hospitals. The MRIs were performed at two of these hospitals, which both had the same model of scanner.

Inclusion criteria: patients aged over 18 with clinically suspected PE referred for CTPA; clinical parameters/patient’s condition that would allow for an additional examination as decided by the patient's physician; written informed consent. Exclusion criteria were: contraindications for MRI; more than 36 hours between CTPA and MRI.

Cases in this study were divided into three groups:

Group 1: Patients referred for CTPA to the radiology department from the emergency department or wards of the hospitals mentioned above who underwent MRI either immediately before the CTPA or within 36 hours after a positive CTPA for PE, depending on availability in the MRI unit. Therefore, most of the patients were examined during the evenings or on weekends. Some of these data (n=22) was published in 2017 as a preliminary study of the method by Nyren et al.[7]

Group 2: Patients referred directly for MRI with clinically suspected acute PE due to contraindications for CTPA and patients taking part in a follow-up study after a confirmed diagnosis of PE with CTPA. Only the cases found to be negative on MRI were included in the present study, all of whom also had a three-month clinical follow-up that was negative for venous thromboembolism (VTE). Part of this material was published as a clinical outcome study in 2019 and the other part as a follow-up study in 2022 by Medson et al.[8], [9]

Group 3: Healthy unmatched volunteers were also scanned to reach a PE prevalence of approximately 20% in the study population.

## 3 Analysis

All CTPAs were read by one radiologist with six years of experience, including one year of cardiothoracic sub-specialty with access to the patient's medical records, including the original clinical CTPA results that are double read as standard in the clinical settings at these hospitals. The emboli were localized according to the system described below using the SECTRA PACS (Sectra AB).

The emboli were localized according to the following:

• Right (R) pulmonary artery, R upper lobe, R middle lobe, R lower lobe.
• Left (L) pulmonary artery, L upper lobe, L lingula, L lower lobe.

• The upper lobes were divided into three segments, middle/lingula in two, and lower lobes into five segments.
• Segmental and subsegmental arteries were assigned to the vascular distribution of the lobar arteries.

When an embolus was diagnosed at a pulmonary or lobar artery level, the vessels distal to that point were not examined. [10], [11] An embolus was diagnosed only on vascular signs, that is, on a complete or partial filling defect and/or railway track sign.[12], [13]

All MRIs were anonymized using the SECTRA PACS. They were then randomized using the online random number generator (random.org) and uploaded to a cloud-based PACS (Purview).

Two radiologists, one with six years of experience, including one year of cardiothoracic sub-specialty(R1), and the other with nine years of experience in general radiology (R2), read the blinded MRI scans separately. No other findings than PE were reported in an attempt to isolate and evaluate the diagnostic accuracy of the method. The time it took to read each case was recorded.

A separate per embolus analysis was also done by comparing the CTPA and MRI for each case side by side to determine if all the emboli diagnosed on CTPA and missed by readers 1 and 2 were visible on MRI. This was done for the repeated protocol developed by us and the standard 2D SSFP sequence, which is the first series out of the five repetitions.

### 3.1 Reference method CTPA

The CTPAs were performed according to the local clinical protocols at the three hospitals (A, B, C) where the patients were recruited from. The image data was then transferred to the SECTRA PACS, where they were analyzed with 0.625 mm slice thickness in multiplanar reconstructions (MPR). The thin slices were unavailable in two cases, which were subsequently analyzed at 5 mm thickness.

### 3.2 Magnetic resonance imaging

All MRI exams were made on a 1.5 tesla scanner (Magnetom Aera, Siemens Healthcare). The protocol used was a standard 2D SSFP sequence with no contrast agent, under free-breathing and without respiratory or cardiac gating repeated five times, resulting in each slice position being imaged five times to capture the vessels at different breathing/cardiac phases. This resulted in approximately 500 images in each orthogonal plane (axial, 600; sagittal, 450; and coronal, 450). Images were then sorted by anatomical position, resulting in five images at various breathing/cardiac phases for each anatomical position. Acquisition time, 9 minutes and 34 seconds (axial 3:50 and sagittal/coronal 2:52).

Parameters were according to the following: a dorsal 32-element spine matrix coil integrated into the table, and a ventral 18-element body matrix coil were used; flip angle, 70°; field of view, 450 mm; matrix size, 256 ×256 voxels; reconstructed voxel size, 0.9 ×0.9 ×4.5 mm^3^; echo time, 1.2–1.3 ms; Repetition time, 2.8–2.9 ms; slice thickness, 4.5 mm; overlap, −2.7 mm (60%).

### 3.3 Statistical analysis

To evaluate the agreement between the two readers, we used both percents of agreement and Cohen’s kappa statistic with its 95%-confidence interval. Cohen’s kappa values were interpreted in the following way; kappa <0.20, poor agreement; kappa = 0.21–0.40, fair agreement; kappa = 0.41–0.60, moderate agreement; kappa = 0.61–0.80, good agreement; kappa = 0.81–1.00, very good agreement.[14] We also calculated the sensitivity; specificity; positive predictive value (PPV); negative predictive value (NPV), and accuracy, where the result of MRI analysis was compared to the gold standard imaging modality for pulmonary embolism CTPA. All analyses were carried out using MedCalc (2022 MedCalc Software Ltd) and the SAS system (the SAS system 9.4 for Windows, SAS Institute Inc.).

## 4 Results

**Fig. 1:**
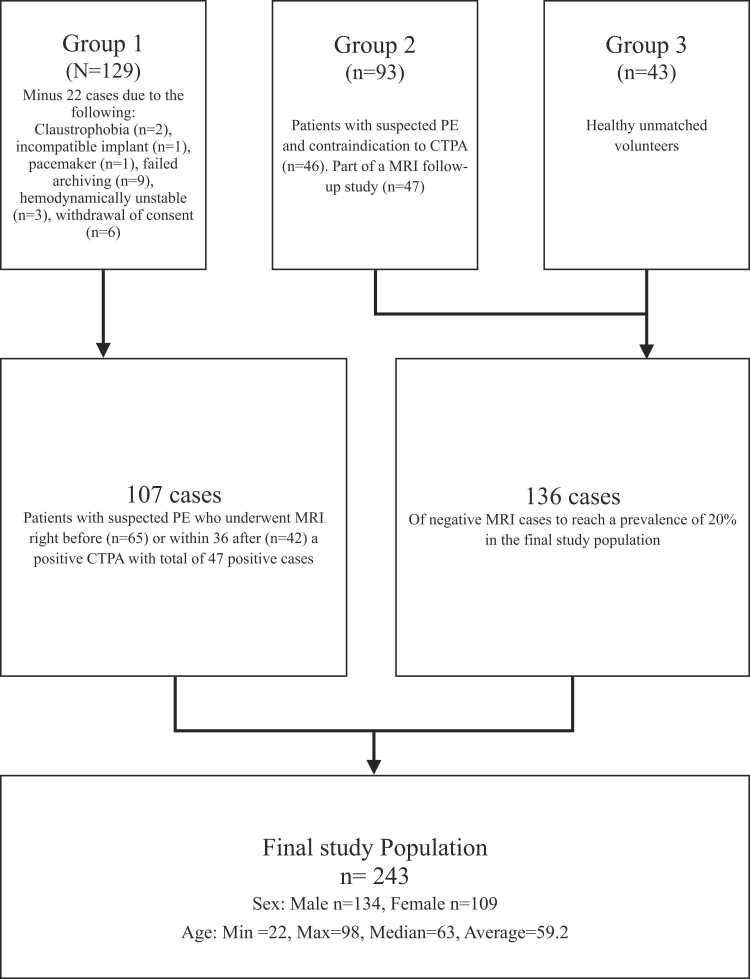
Patient inclusion Flowchart.

MRI scans were consented or completed, with a final study population of 243 remaining (Fig. 1.). 109 females and 134 males, with an average age of 59.2 years (median 63, min 22, max 98). Forty-seven patients were diagnosed with PE on CTPA, which resulted in a prevalence of 19.3%. All the CTPA and MRI scans qualified for having diagnostic quality.

**Fig. 2:**
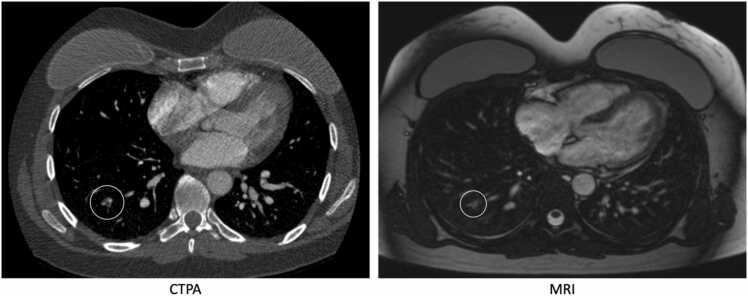
45 years old female, 16 hours and 20 minutes between CTPA and MRI. Missed subsegmental emboli in RLL on MRI by R1 and R2.

### 4.1 Group 1

One hundred and seven patients were included in this group, 49 females and 58 males, with a mean age of 61.7 (median 65, min 22, max 87). All were referred to the radiology department for suspected acute PE from the emergency department or a ward.

Sixty-five of these patients underwent MRI before the CTPA with a mean wait time of 1:09 (hour:minute) (minimum 00:33; maximum 5:10) between the examinations. There were 10 positive cases in this subgroup.

Forty-two patients underwent MRI within 36 hours after a positive CTPA with a mean time of 22:50 (minimum 2:19; maximum 34:50). The mean time between the two examinations for all the patients in this group was 15:52. This was done to increase the number of positive cases in the study population. All of the patients in this subgroup were already on or had been started on anticoagulation before the MRI exam.

**Fig. 3:**
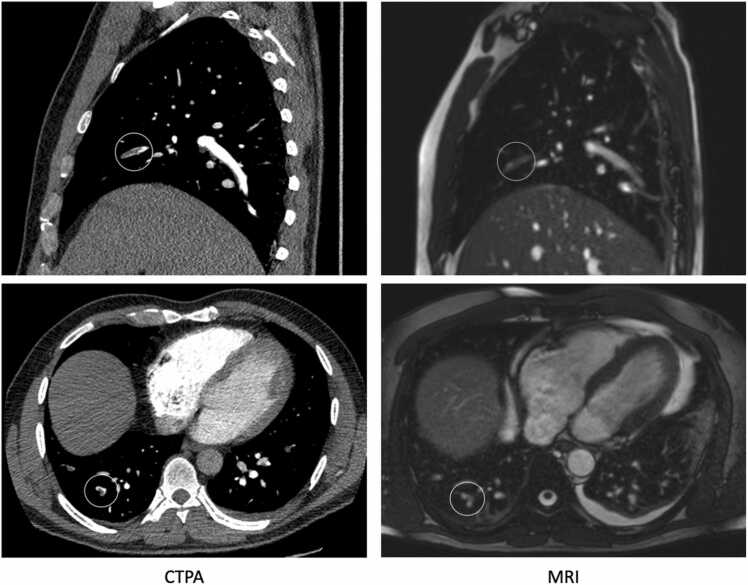
39 years old male, 17 hours between CTPA and MRI. Missed subsegmental emboli in RML and RLL on MRI by R2.

### 4.2 Group 2

This included 93 cases, 55 females and 38 males, who underwent MRI only due to suspected acute PE and contraindications to CTPA (n=46) such as renal failure, severe contrast allergy, pregnancy, or as part of a PE follow-up study (n=47). These patients all had a negative MRI result and a negative 3-month clinical follow-up for VTE with a mean age of 63.2 (median 66, minimum 28, maximum 98) years.

### 4.3 Group 3

This included 43 unmatched healthy volunteers, 22 females and 21 males, with a mean age of 44.6 (median 45, minimum 26, maximum 66) years.

The number of emboli diagnosed on CTPA at different levels was as follows: Proximal, 61, segmental, 113, subsegmental, 32 (Table 1). Six patients had proximal only, five patients had segmental only, and seven had subsegmental PE only. Twenty-five patients had both proximal and distal (segmental and subsegmental) PE. The prevalence of isolated subsegmental emboli in the present study was 15% which falls within the range of 7–38% presented in the literature.[15]The analysis at both the patient and the artery/embolus levels in reference to CTPA are presented in Table 2.

**Table 1:** Detected emboli at different artery-level by CTPA, MRI, R1, and R2.

	Right										Left									
	Pulmonary artery	Upper lobe	Seg	Sub	Middle lobe	Seg	Sub	Lower lobe	Seg	Sub	Pulmoary artery	Upper lobe	Seg	Sub	Lingula	Seg	Sub	Lower lobe	Seg	Sub
CTPA	6	8	14	3	13	4	4	15	42	14	8	1	12	2	7	6	4	3	35	5
R1	6	8	9	1	13	3	1	15	41	2	8	1	9	0	6	5	0	3	34	1
R2	6	8	6	0	12	4	1	15	42	1	8	1	8	0	6	2	0	3	32	0
MRI, Repeated Protocol	6	8	12	2	13	3	2	15	42	10	8	1	11	2	7	6	0	3	35	4
MRI, Standard Sequence	6	8	7	0	13	3	1	15	41	2	8	1	7	0	6	3	0	3	26	0

**Table 2:** Detected emboli at different levels with CTPA, MRI repeated protocol, MRI standard 2D SSFP sequence and by readers R1 and R2.

	Patient-level	Proximal arteries	Segmental arteries	Subsegmental arteries
CTPA	47	61	113	32
R1, MRI	42 (89%)	60 (98%)	101 (89%)	5 (16%)
R2, MRI	41(87%)	59 (97%)	94 (83%)	2 (6%)
MRI, Repeated protocol	46 (98%)	61 (100%)	109 (96%)	20 (63%)
MRI, Standard sequence	42 (89%)	60 (98%)	86 (76%)	3 (9%)

The number in parenthesis represents the percentage of detected emboli with MRI in reference to CTPA.

Reader 1 = R1, Reader 2 = R2

In eleven cases, the localization of the diagnosed emboli differed between readers R1 and R2; one reader had diagnosed an embolus as segmental or subsegmental, whereas the other reader had diagnosed the same embolus as lobar or segmental, respectively.

### 4.4 Reader 1

Reader one (R1) detected 60/61 at proximal, 101/113 at segmental, and 5/32 at subsegmental arteries. The mean time for reading the studies was 3:25 (min 1:10, max: 5:22). Five patients with isolated subsegmental PE would have been misdiagnosed by R1. At the patient level, the sensitivity for R1 is 89.36%, and the specificity is 100%. At the embolus level, the sensitivity for R1 decreases to 80.58%.

### 4.5 Reader 2

Reader two (R2) detected 59/61 at proximal, 94/113 at segmental, and 2/32 at subsegmental arteries. The mean time for reading the studies was 2:32 (min 1:47, max: 3:35). Six patients with isolated subsegmental PE would have been misdiagnosed by R2, who also had two false-positive emboli at the segmental level in the right middle and lower lobe in patients with multiple other emboli at lobar and segmental levels. At the patient level, the sensitivity for R2 was 87.23%, and the specificity was 100%. At the embolus level, the sensitivity for R2 decreased to 75.24%.

#### 4.5.1 Side by side per embolus comparison of CTPA and MRI (repeated protocol)

In the side-by-side per embolus comparison of the positive CTPAs and MRIs, 61 of 61 proximal, 109 of 113 segmental, and 20 of 32 subsegmental emboli were visible on MRI. The average time between CTPA and MRI for the non-visible emboli at segmental and subsegmental levels on MRI was 11:11 (minimum 34, maximum 32:41) and consisted of six women and six men with a mean age of 56.4 (minimum 37, maximum 88) years.

One patient with an isolated subsegmental embolus had a false negative MRI, as it was not visible in the side-by-side comparison of the two modalities. The patient was a 77-year-old female with an isolated subsegmental embolus in the right upper lobe. The time between the MRI and CTPA was 13:50.

At the patient level in a side-by-side comparison, the sensitivity of the method was 97.87%, specificity 100%, PPV 100%, NPV 99,49%, and accuracy of 99.59%. At the embolus level, the sensitivity of the method decreased to 92.23% (Table 3).

**Table 3:** Statistics for MRI repeated protocol, MRI standard 2D SSFP sequence, R1, R2 and per embolus in reference to CTPA.

	Sensitivity	Specificity	PPV	NPV	Accuracy	Cohens kappa
R1, patient-level	89.36	100	100	97.51	97.94	0,88
R2, patient-level	87.23	100	100	97.03	97.53	
MRI, Repeated protocol, patient-level	97.87	100	100	99.49	99.59	
MRI, Standard sequence, patient-level	89.36	100	100	97.51	97.94	
R1, Embolus-level	80.58					
R2, Embolus-level	75.24					
MRI, Repeated protocol, Embolus-level	92.23					
MRI, Standard sequence, Embolus-level	72.81					

Reader 1 = R1, Reader 2 = R2

#### 4.5.2 Side by side per embolus comparison of CTPA and MRI (standard sequence)

In the side by side per embolus comparison of the positive CTPAs and MRIs, 61 of 61 proximal, 86 of 113 segmental, and 3 of 32 subsegmental emboli were visible on MRI.

Five patients with an isolated subsegmental embolus had false negative MRI, as it was not visible in the side-by-side comparison of the two modalities.

At the patient level in a side-by-side comparison, the sensitivity of the method is 89.36%, specificity 100%, PPV 100%, NPV 97.51%, and accuracy of 97.94%. At the embolus level, the sensitivity of the method decreased to 72.81% (Table 3).

The number and localization of the emboli, the time between the CTPA and MRI, the age, and sex of all the patients missed by MRI, and the readers R1 and R2 are shown in Table 4.

**Table 4:** Details of patients and the emboli missed by R1, and R2.

Patient	Age	Sex	Time Hours: min	Missed by	Location
1	55	M	8:41	MRI*, R1 & R2	LLL, Subsegmental RLL, Subsegmental
2	64	F	22:57	R1 & R2	RLL, Subsegmental
3	45	F	16:20	R1 & R2	RLL, Subsegmental
4	48	F	1:04	R1 & R2	RLL, Subsegmental
5	39	M	17:00	R2	RML, Subsegmental RLL, Subsegmental
6	77	F	13:50	MRI, R1 & R2	RUL, Subsegmental

The time between examinations is given in hours:minutes. Reader 1 = R1, Reader 2 = R2, RLL = Right Lower Lobe, LLL = Left Lower Lobe, RML = Right Middle Lobe,

RUL = Right Upper Lobe. * = MRI missed only RLL

The inter-reader agreement between the two radiologists R1 and R2 at patient level using Cohen's kappa was 0.88.

## 5 Discussion

The principal finding of the present study is that our MRI method has a high diagnostic accuracy for acute PE at proximal and segmental levels. It is a relatively fast, non-contrast protocol and technically easy both for the patients and the MRI technicians.

Two of the large MRI studies regarding PE, namely PIOPED III and IRM-EP showed that the use of magnetic resonance angiography (MRA) with gadolinium contrast agent resulted in technically inadequate studies in up to 30% of the cases.[6], [16] Furthermore, there are safety considerations regarding gadolinium. It is contraindicated in chronic kidney disease and in pregnant patients. It is also associated with nephrogenic systemic fibrosis.[17]

The protocol in the present study is based on 2D SSFP with no need for a gadolinium contrast agent. It is a fast sequence using single-shot acquisition, which makes it less susceptible to motion artifacts and so there is no need for breath-hold, respiratory, or cardiac gating. These factors contribute to make the examination less technically demanding compared to MRA, which in turn leads to a very low number of non-diagnostic studies.

Several studies have used 2D SSFP alone or in combination with other sequences for the detection of acute PE.

The following three studies have shown relatively high sensitivity, which is in line with the present study. In 2004, Kluge et al. demonstrated that the 2D SSFP sequence had a sensitivity of 83% and a specificity of 100% (n = 39) in reference to the ventilation/perfusion (V/Q) scan. They concluded that its sensitivity and specificity in reference to V/Q and MRA were sufficient to allow the diagnosis of central, lobar, and segmental PE, and that the method is feasible for PE diagnosis in an emergency setting.[18] Another study, made by Pasin et al. in 2017 included 93 patients and demonstrated that the non-contrast 2D SSFP sequence has a sensitivity of 85%, and specificity of 98.6%, with a kappa value of 0.87 at the patient level, and a slight decrease when calculated at the embolus level. They concluded that the non-contrast MRI sequence demonstrated good accuracy and that MRI was as capable as CTPA in detecting life-threatening conditions.[19] The highest sensitivity in detecting acute PE with an 2D SSFP sequence was demonstrated by Nyren et al. in 2017, using the same repeating 2D SSFP protocol as the present study in a cohort of 33 patients. They showed a sensitivity of 90–93% for the two readers and a kappa value of 0.97.[7]

The main difference in the protocol used in the present study and that by Nyren et al. in comparison to the other studies mentioned above is the use of a repeating protocol, which we believe may increase the ability of the reader to detect the emboli for the following reasons:

1. The emboli are visible in multiple sequential images when scrolling through the stacks, making them more detectable to the radiologist.
  Comparing the repeated protocol with the standard sequence shows that some of the emboli will be missed because the vessels are not imaged/captured since the examination is done under free breathing and without cardiac or respiratory gating. (Table 3 and Fig. 4)

**Fig. 4:**
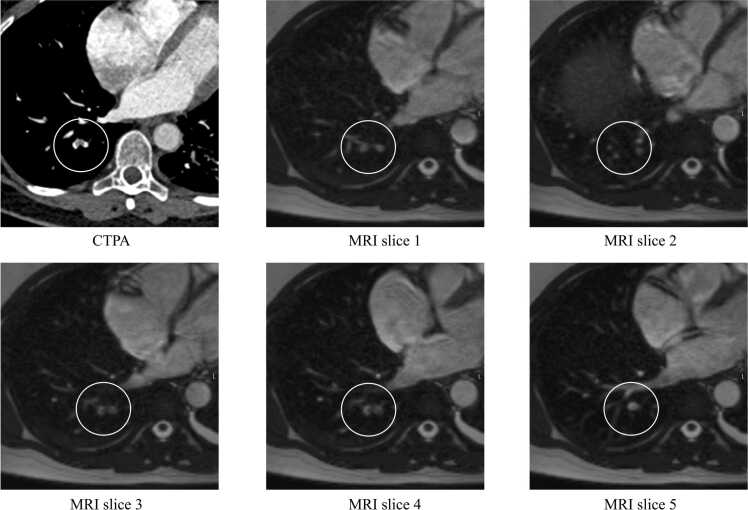
As shown in these figures the embolus is only visible in slices 3 and 4, that is, in only 2 out of the 5 MRI images from the same slice position.
2. Following the vessels while scrolling through the stacks feels subjectively easier and more like CT stacks, according to the radiologists analyzing the studies, thereby making it more familiar. This could be explained by the number of images in the MRI stacks, which are approximately the same as in CTPA.

This was also demonstrated by Nordgren et al. in 2017, who found that radiology residents who were used to diagnosing PE on CTPA could quickly learn to read these MRI scans.[20]

Another interesting point is that R2 of the present study had no previous experience with this method and still demonstrated excellent detection accuracy. The diagnostic accuracy in PE diagnostics is crucial in the clinical setting with decisions on whether a patient should receive anticoagulation treatment or not. Although the method in the present study lacks the sensitivity of CTPA, especially at the subsegmental level and takes longer time, several points support its use.

Available data clearly shows an increase in the number of diagnosed PE since the emergence of CT, without any significant changes in mortality. Furthermore, the importance of sub-segmental pulmonary embolisms (SSPE) is still an ongoing debate, with conflicting results being presented.[21], [22], [23] These conflicts imply a knowledge gap regarding the SSPE, which will be bridged hopefully with the ongoing SAFE-SSPE study.[24] Meanwhile, the latest recommendations by The American College of Chest Physicians (CHEST) from 2016 are that in patients with SSPE and with a transient VTE risk factor and no proximal DVT, surveillance is suggested over anticoagulation.[25] With that in mind, it is important to note that all the patients misdiagnosed in the present study had SSPE only. This is an essential point in favor of this non-contrast 2D SSFP.

Another important supporting aspect of this MRI method is that, like CT, it can diagnose other cardiothoracic conditions such as pericardial and pleural effusion, aortic dissection, pulmonary opacities, bleeding, and also assess for heart strain and right ventricular / left ventricular (RV/LV) ratio which would not be possible with V/Q scan that is the second line method. [7], [8], [19] This MRI method also open the possibility of extending the imaging to the pelvis and legs for DVT assessment. [26]

There are additional research efforts aimed at the innovation of MRI sequences to enhance the speed and clarity of pulmonary embolism (PE) visualization. To our knowledge, these are primarily smaller-scale technical feasibility studies. [27], [28]

## 6 Strengths and limitations

The strength of the present study is the relatively large study population and the large number of MRIs made before the CTPA.

We lack CTPA in some of the negative controls, however, a negative follow-up for three months was considered sufficient for a negative diagnosis. Another limitation might be the fact that we added healthy volunteers, which was done solely to attain a 20% prevalence, simulating the unselected population of CTPAs read by radiologists at our institution. Important to point out that incorporating this group did not alter the patient-level results for sensitivity or specificity, as false positives were zero, and true positives and false negatives were unaffected by these groups.

There was also the limitation that not all patients had the MRI before the CTPA, leaving the possibility that an embolus dissolved between the two examinations since almost all patients were put on anticoagulation by the time of examination. The two points mentioned above are part of the heterogeneity of the study population, which we consider an important limitation of the present study.

Having only one reader for the CTPA should also be mentioned as a limitation.

## 7 Conclusions

The non-contrast repeated 2D SSFP under free-breathing and without respiratory or cardiac gating can reliably be used for the diagnosis of acute PE at proximal and segmental artery levels. Considering the ease of acquisition and read, relatively short exam time, increased availability/accessibility of MRI scanners, and the method's diagnostic performance as shown in the present study, we believe this method could greatly benefit patients with contraindications to CTPA.

## Funding statement

This study has received funding from The Swedish Heart and Lung Foundation, The Swedish Society of Medicine, and the Stockholm City Council.

## Ethical statement

This study was approved by the local ethics committee in Stockholm. Written informed consent was obtained from all the research objects.

## CRediT authorship contribution statement

**Peter Lindholm:** Writing – review & editing, Supervision, Project administration, Methodology, Funding acquisition, Conceptualization. **Alexander Fyrdahl:** Writing – review & editing, Software, Methodology. **Roberto Vargas Paris:** Methodology, Data curation, Conceptualization. **Koshiar Medson:** Writing – original draft, Project administration, Methodology, Formal analysis, Data curation. **Eli Westerlund:** Writing – review & editing, Supervision, Project administration, Data curation. **Sven Nyren:** Methodology, Investigation, Data curation, Conceptualization. **Peder Wiklund:** Writing – review & editing, Formal analysis.

## Declaration of Competing Interest

The authors declare that they have no known competing financial interests or personal relationships that could have appeared to influence the work reported in this paper
